# Simple fabrication of electrochemical sensor based on integration of dual signal amplification by the supporting electrode and modified nanochannel array for direct and sensitive detection of vitamin B_2_

**DOI:** 10.3389/fnut.2024.1352938

**Published:** 2024-03-15

**Authors:** Yafei Wu, Zhuxuan Shi, Junjie Liu, Tao Luo, Fengna Xi, Qi Zeng

**Affiliations:** ^1^Guangxi Medical University Cancer Hospital, Guangxi Medical University, Nanning, China; ^2^School of Chemistry and Chemical Engineering, Zhejiang Sci-Tech University, Hangzhou, China

**Keywords:** electrochemical sensor, easy fabrication, dual signal amplification, nanochannel array, vitamin B

## Abstract

Development of simple and reliable sensor for detecting vitamin content is of great significance for guiding human nutrition metabolism, overseeing the quality of food or drugs, and advancing the treatment of related diseases. In this work, a simple electrochemical sensor was conveniently fabricated by modification a carbon electrode with vertically-ordered mesoporous silica film (VMSF), enabling highly sensitive electrochemical detection of vitamin B_2_ (VB_2_) based on the dual enrichment of the analyte by the supporting electrode and nanochannels. The widely used glassy carbon electrode (GCE), was preactivated using simple electrochemical polarization, The resulting preactivated GCE (p-GCE) exhibited improved potential resolution ability and enhanced peak current of VB_2_. Stable modification of VMSF on p-GCE (VMSF/p-GCE) was achieved without introducing another binding layer. The dual enrichment effect of the supporting p-GCE and nanochannels facilitated sensitive detection of VB_2_, with a linear concentration ranged from 20 nM to 7 μM and from 7 μM to 20 μM. The limit of detection (LOD), determined based on a signal-to-noise ratio of three (S/N = 3), was found to be 11 nM. The modification of ultra-small nanochannels of VMSF endowed VMSF/p-GCE with excellent anti-interference and anti-fouling performance, along with high stability. The constructed sensor demonstrated the capability to achieve direct electrochemical detection of VB_2_ in turbid samples including milk and leachate of compound vitamin B tablet without the need for complex sample pretreatment. The fabricated electrochemical can be easily regenerated and has high reusability. The advantages of simple preparation, high detection performance, and good regeneration endow the constructed electrochemical sensor with great potential for direct detection of small molecule in complex samples.

## 1 Introduction

Vitamins are essential micronutrients for the human body, playing a crucial role in maintaining normal physiological functions. Different vitamins are involved in various physiological processes. For instance, Vitamin B_2_ (VB_2_), also known as riboflavin, is the sole precursor for the biosynthesis of the coenzymes flavin mononucleotide (FMN) and flavin adenine dinucleotide (FAD) (1–3). Generally, a deficiency in VB_2_ can lead to metabolic disorders, delayed growth in children, skin and mucous membrane lesions, and even diseases such as cancer. On the other hand, excessive intake of VB_2_ may cause phototoxic damage to exposed tissues or DNA degradation (4). In Addition, VB_2_ also has potential applications in medical treatments, such as photodynamic therapy for destroying tumor tissues, treating cardiovascular diseases (such as stroke), and exhibiting antibacterial properties (5). However, VB_2_ cannot be synthesized by the human body and cannot be stored for an extended period within the body (1, 2). Therefore, humans and all animals need to continuously obtain VB_2_ from food to sustain the metabolic processes involving the abundant coenzymes of VB_2_. As a water-soluble vitamin, VB_2_ is widely present in foods such as organ meats, dairy products, whole grains, and egg yolks. However, due to its photosensitivity, a decrease in its content is often considered a sign of food deterioration in food quality monitoring (6–9). Consequently, the development of simple and accurate methods for detecting VB_2_ content is of great significance in guiding human nutritional metabolism, supervising the quality of food or drugs, pharmacological research, clinical diagnosis, and the treatment of related diseases.

The current methods for detecting VB_2_ mainly based on fluorescence spectroscopy (10, 11), chemiluminescence (12), high-performance liquid chromatography (HPLC) (13), and electrochemical sensors (14). However, fluorescence detection is susceptible to background signals from matrix. Chemiluminescence requires high sample purity. Although HPLC is suitable for the analysis of mixtures, it often requires complex pre-treatment, long analysis time and expensive instrument. Electrochemical sensors have advantages such as rapid detection, high sensitivity, ease of miniaturization, and simplicity of instrumentation, making them widely used in analysis of pharmaceutical, food, and clinical samples (15–17). As a water-soluble substance with three connected aromatic rings and multiple hydroxyl groups, VB_2_ is electroactive which undergoes a redox reaction on the electrode involving two protons and two electrons, generating electrochemical signals for detection (18, 19). For instance, Gizaw et al. developed a polyglutamic acid-ZnO nanoparticle modified electrode for detecting VB_2_ in non-alcoholic beverages and milk samples (20). Lucas et al. constructed an alkylthiol-modified gold electrode, achieving sensitive detection of VB_2_ through nonpolar interactions between the alkylthiol end group and VB_2_ (21). However, some electrode fabrication steps in these studies are intricate. Moreover, the complex matrix in real samples can affect the analysis. On the one hand, large molecules in samples, such as proteins, can nonspecifically adsorb and occupy active sites on the electrode surface, leading to electrode fouling. On the other hand, other electroactive compounds in the samples can significantly interfere with the signal, reducing the sensitivity, accuracy, and repeatability of electrochemical sensing. Therefore, establishing a simple electrode modification method and enhancing the anti-fouling/anti-interference abilities are highly desirable.

Introducing nanomaterials onto electrodes can significantly enhance electrode performance and broaden its applications (22–25). Among, utilizing porous films to modify electrodes not only optimizes electrode performance but also contributes to its widespread applications in sensing, electrocatalysis, and energy storage (26, 27). Firstly, the porous structure provides a larger surface area, increasing the contact area between the electrode and electrolyte, enhancing the reactivity of electrode (28). Secondly, the porous structure helps improve the mass transfer performance, facilitating more efficient transfer of protons or ions between the electrolyte and electrode, improving the reaction kinetics on electrode (29). Additionally, the selectivity of the porous structure can be enhanced by selective controlling pore size and porosity, allowing precise recognition and transfer of specific ions or molecules (30, 31). Porous films can also serve as carriers to support the fixation of catalysts or other functional materials, enhancing their stability (32–35). Among different porous films, vertically-ordered mesoporous silica film (VMSF) possesses unique structures and properties, garnering significant attention in separation and catalysis fields (36–40). Specifically, VMSF exhibits a highly ordered nanochannel array with high-density (up to 10^12^/cm^2^), tunable nanochannel diameter (typically ranged from 2 nm to 11 nm), adjustable film thickness (usually 50–200 nm), and excellent chemical stability (41). Due to the ultra-small nanochannels relative to the double-layer thickness or Debye length, VMSF demonstrates selective permeability based on charge and molecular size (42–44). For instance, the high surface area of VMSF can significantly enhance detection sensitivity by electrostatic and hydrogen bonding interactions, allowing for the enrichment of target analytes or probes (45–49). Simultaneously, VMSF can mitigate interference from coexisting components in complex matrices through size exclusion and electrostatic repulsion effects (50). For instance, VMSF possesses an insulating silica structure. The ultra-small nanochannels of VMSF can size-exclude large molecules (e.g., proteins, DNA and starch) or particles (e.g., cells, particulate matter), preventing contamination of the underlying electrode (51). On the other hand, the negatively charged surface resulting from ionization of silanol groups can repel common electroactive interferents in complex samples (52). Thus, VMSF-modified electrodes exhibit excellent anti-contamination and interference resistance, holding potential for direct electroanalysis in complex samples. Due to the inert silica structure of VMSF, the selective detection of many electroactive substances by VMSF-based sensors still relies on the electrochemical activity and potential resolution of the underlying electrode. However, except for indium tin oxide (ITO) electrodes, carbon electrodes or noble metal electrodes (gold, platinum) cannot directly achieve stable binding with VMSF. However, ITO electrodes often exhibit a high overpotential when detecting organic electrochemical small molecules. Enhancing the potential resolution and electroactivity of electrodes such as carbon electrodes through convenient electrode modification methods and achieving stable modification of VMSF can effectively expand the application of VMSF-based electrodes in the electrochemical detection of small molecules in complex matrices.

In this work, an electrochemical sensor was conveniently fabricated through a simple and easy-to-operate method, achieving direct and highly sensitive detection of VB_2_ in complex samples. The commonly used carbon-based electrode, glassy carbon electrode (GCE), was employed as the supporting electrode. Simple electrochemical polarization was employed to pretreat GCE (p-GCE), endowing the supporting electrode with high peak current, good potential resolution, and the ability to directly and stably bind with vertically-ordered mesoporous silica film (VMSF/p-GCE). Both p-GCE and VMSF nanochannels can enrich VB_2_, achieving dual signal amplification. Combining the potential resolution of p-GCE with ultra-small nanochannel array of VMSF, VMSF/p-GCE exhibits good selectivity, high anti-fouling performance and stability, enabling direct electrochemical detection of VB_2_ in turbid samples without the need for complex sample pretreatment. The sensor constructed in this study has advantages including simple preparation method, high sensitivity, good selectivity and great potential in direct electroanalysis of complex samples.

## 2 Materials and methods

### 2.1 Chemicals and materials

The chemicals utilized in this work were all of analytical purity and required no additional treatment. Specifically, bovine serum albumin (BSA), cetyltrimethyl ammonium bromide (CTAB, 99%), vitamin B_2_, and vitamin B_6_ were procured from Maclin Biochemical Technology Co., LTD (Shanghai, China). Sodium hydrogen phosphate heptahydrate (Na_2_HPO_4_•7H_2_O), sodium dihydrogen phosphate (NaH_2_PO_4_), tetraethyl orthosilicate (TEOS, 98%), potassium hydrogen phthalate (KHP), potassium ferricyanide (K_3_Fe(CN)_6_), sodium hydroxide (NaOH), hexammine ruthenium (III) trichloride (Ru(NH_3_)_6_Cl_3_), starch, glucose, ascorbic acid (AA), dopamine (DA), and uric acid (UA) were acquired from Aladin Reagent Co., LTD (Shanghai, China). Ethanol, sodium nitrate (NaNO_3_), sodium chloride (NaCl), calcium chloride (CaCl_2_), potassium chloride (KCl), ferric chloride (FeCl_2_), and magnesium chloride (MgCl_2_, 95%) were sourced from Gaojing Fine Chemical Co., LTD (Hangzhou, China). Concentrated hydrochloric acid was obtained from Shuanglin Chemical Reagent Co., LTD. The ultrapure water (18.0 MΩ•cm) used in the experiments was prepared by Mill-Q Systems (Millipore). Multivitamin B tablets were acquired from the local pharmacy store (Hangzhou, China). Milk samples were obtained from the local supermarket (Hangzhou, China).

### 2.2 Characterization and instrumentations

The morphology and thickness of VMSF were characterized using an HT7700 transmission electron microscope (TEM) from Hitachi, Japan. The sample preparation involved careful scraping off the VMSF layer from p-GCE using a knife and ultrasonical dispersing it in anhydrous ethanol. The resulting supernatant drops were coated onto a copper grid, dried, and then observed in the TEM chamber at an accelerated voltage of 200 kV. All electrochemical tests were conducted on an Autolab PGSTAT302N electrochemical workstation (Metrotron, Switzerland). The three-electrode system was employed, with the reference electrode being Ag/AgCl (saturated using KCl), the counter electrode being platinum wire or disk (2 cm × 4 cm), and the working electrode being either p-GCE or VMSF/p-GCE. The scan rate in the cyclic voltammetry (CV) test was set at 100 mV/s. For differential pulse voltammetry (DPV) measurements, the parameters including step potential (5 mV), pulse amplitude (25 mV), pulse time (0.05 s), and time interval (0.2 s) were set.

### 2.3 Preparation of p-GCE

Initially, GCE underwent successive polishing with 0.5 μm, 0.3 μm, and 0.05 μm alumina powder. Following the polishing step, GCE surface was subjected to ultrasonic cleaning in ethanol and ultrapure water for 1 min each to remove residual alumina powder, ensuring a cleaner electrode surface. The newly polished GCE was activated using electrochemical polarization under static conditions. Briefly, a phosphate buffer solution (PBS, 0.1 M, pH = 5) served as the electrolyte. The electrochemical polarization involved both anodic oxidation and cathodic reduction (49). Anodic oxidation was carried out using a constant voltage of +1.8 V for 5 min. Subsequently, the obtained electrode underwent cathodic reduction at −1.0 V for 1 min, resulting in the electrochemically pre-activated GCE, referred to as p-GCE.

### 2.4 Fabrication of VMSF/p-GCE

The modification of VMSF on p-GCE electrode was achieved through an electrochemical-assisted self-assembly (EASA) method (53). Initially, a precursor solution was prepared by weighing 1.585 g of CTAB and adding it to a mixed solution consisting of 20 mL ethanol and 20 mL NaNO_3_. The mixture was stirred until completely dissolved. Subsequently, 3,050 μL of TEOS was added as the siloxane source, and the solution was stirred at room temperature for 2.5 h. For VMSF growth, the three-electrode system was immersed in the obtained precursor solution and a constant current (−0.7 mA/cm^2^) was applied on p-GCE for 10 s. Then, the resulting electrode was promptly removed and immersed in ultrapure water for thorough washing. After aging overnight at 80°C, the electrode with surfactant micelles (SM) blocking the nanochannels was obtained, denoted as SM@VMSF/p-GCE. Immersing it in a hydrochloric acid/ethanol solution to remove SM from the nanochannels resulted in an electrode modified with an array of open nanochannels (VMSF/p-GCE).

### 2.5 Electrochemical detection of VB_2_

When detecting VB_2_ with VMSF/p-GCE, a supporting electrolyte solution of PBS (0.1 M, pH = 7.0) was used. Different concentrations of VB_2_ were introduced into the electrolyte solution, and the signals generated by the electrochemical oxidation reaction of VB_2_ on the electrode were determined using the DPV method. For real sample analysis, VB_2_ in milk or composite vitamin B tablets was determined. For milk samples, the samples were diluted 1,000 times with the supporting electrolyte solution before testing. For composite vitamin B tablets, the tablets were firstly ground into powder using a mortar and pestle, and 0.787 g of the powder was dispersed in ultrapure water (2 mL). Then, VB_2_ content in the diluted milk or tablet leachate was determined using the standard addition method.

## 3 Results and discussion

### 3.1 Electrochemical preactivation of GCE and stable integration of VMSF on electrode surface

**Figure 1:**
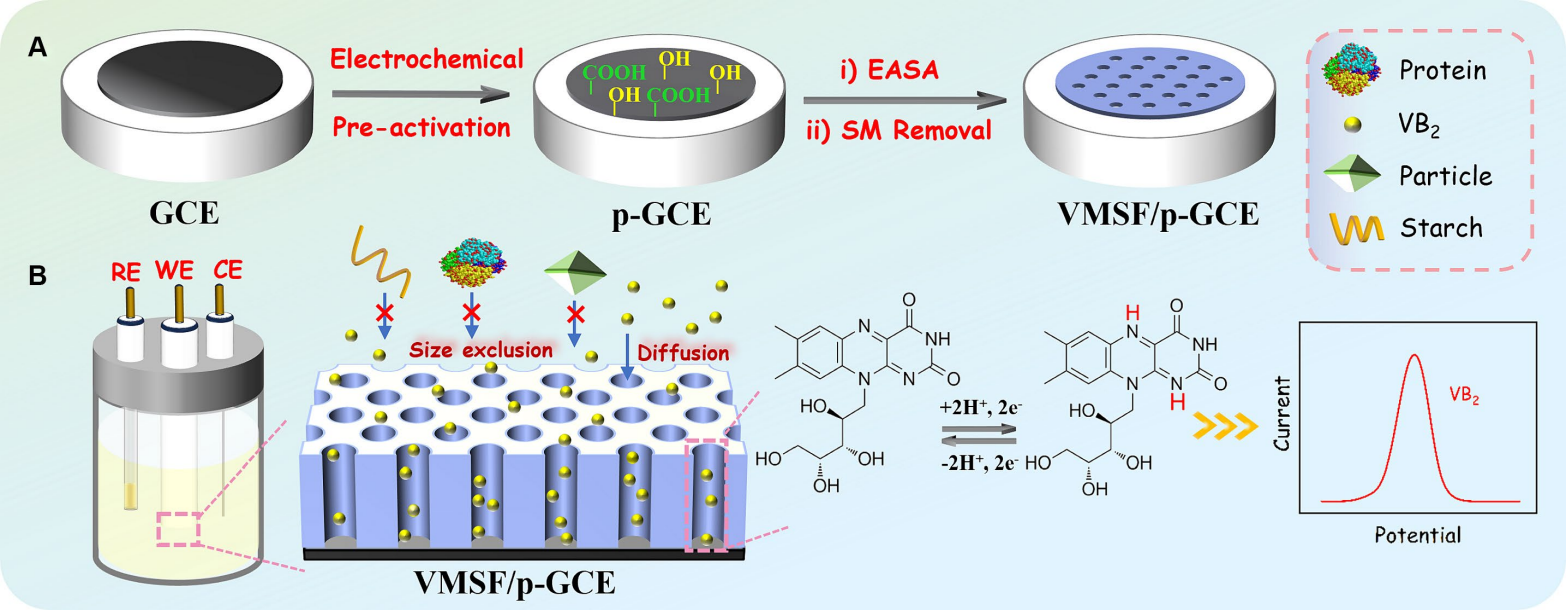
Illustration for the fabrication of VMSF/p-GCE for electrochemical detection of VB_2_.

In this work, a simple electrochemical sensor was efficiently prepared for direct electrochemical detection of VB_2_ by integrating VMSF with highly active p-GCE. As illustrated in Figure 1, the widely used GCE was chosen as the supporting electrode. However, GCE can not stably bind with VMSF and its electrochemical activity should be improved. Activating carbon-based electrodes through electrochemical polarization is a simple, green method for preparing highly active electrodes. This method requires no complex chemicals or cumbersome procedures and typically involves electrochemical pretreatment of the electrode in a conventional electrolyte solution. Thus, electrochemical preactivation of GCE through electrochemical polarization was performed to obtain p-GCE with high electrochemical activity and ability to stably bind with VMSF. Herein, electrochemical polarization was performed using anodic oxidation and cathodic reduction sequentially in a PBS solution. Specifically, a higher positive potential (+1.8 V) was applied to GCE for anodic polarization, followed by cathodic reduction at a negative potential (−1 V). During the anodic oxidation process, active oxygen radicals generated from water electrolysis oxidize and etch the sp^2^-conjugated carbon on GCE surface, leading to the formation of abundant edge carbon, defects, and oxygen-containing functional groups (54). X-ray photoelectron spectroscopy (XPS) characterization was employed to investigate the change of the surface chemical groups. The atomic percentages of carbon in GCE, the electrode after anodic polarization, and p-GCE are 14.6, 69.9, and 75.7%, respectively. The corresponding oxygen atomic percentages are 14.6, 30.0, and 24.3%. The changed atomic percentages indicates the changed chemical structure on the electrode surface. Supplementary Figure S1 displays high-resolution C1s spectra of GCE, the electrode after anodic polarization, and p-GCE. As shown, all electrodes have four carbon bonds including C-C/C=C(sp^2^ C, 284.4 eV), C-O (285.8 eV), C=O (287.1 eV), and O-C=O (288.6 eV). After anodic polarization, the content of C=O and O-C=O bonds on electrode surface significantly increased, while the content of C-O bonds decreased, suggesting an increased oxidation degree of carbon atoms under high potential. Following the subsequent cathodic reduction, the content of C-O bonds increased, and the content of C=O bonds decreased significantly, indicating the reduction of C=O bonds to C-O bonds. Compared to GCE, the C-O bond content on p-GCE increased from 21.4 to 24.5%, indicating an increase in oxygen-containing groups on the GCE surface after electrochemical polarization. These functional groups serve as active sites, enhancing the adsorption of organic electroactive molecules on electrode and facilitating interfacial electron transfer reactions. Thus, compared to traditional GCE, p-GCE exhibits higher electrochemical and electrocatalytic activity.

**Figure 2:**
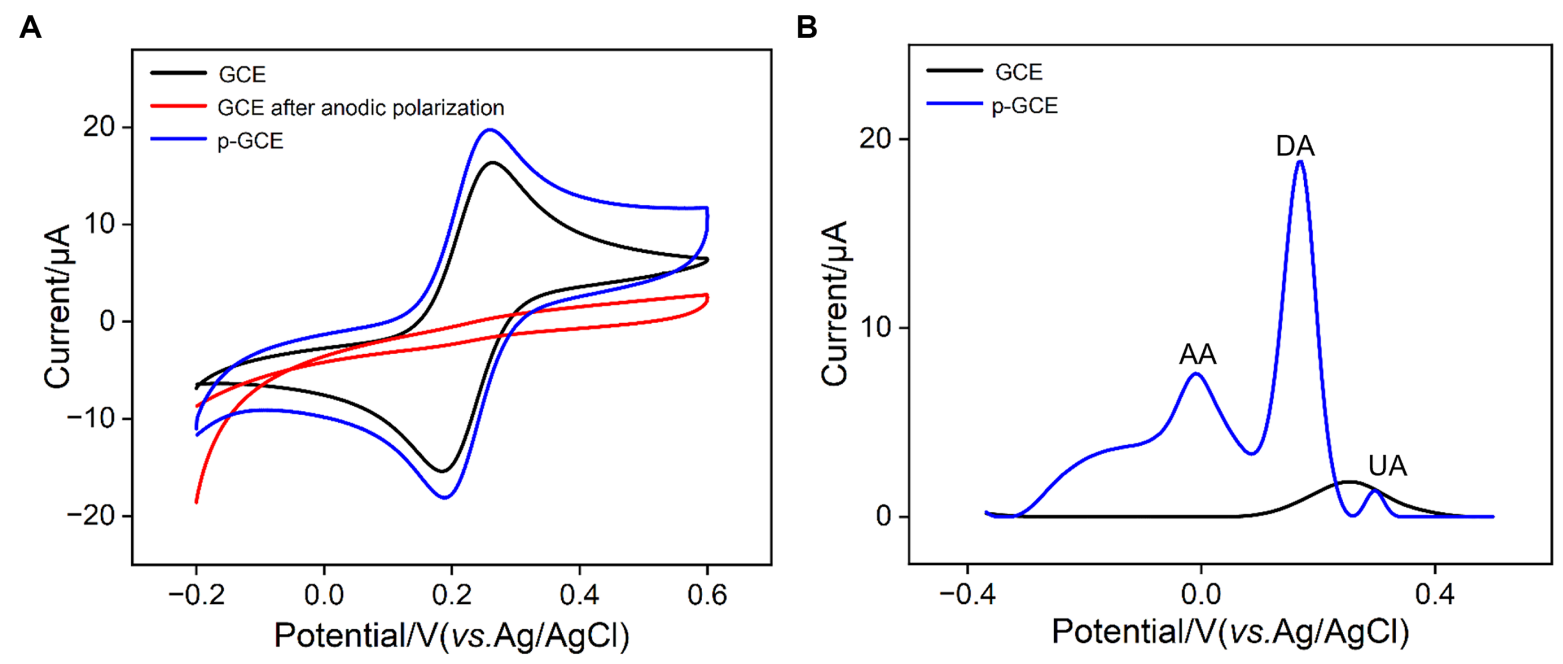
**(A)** CV curves obtained on different electrodes in 1 mM Fe(CN)_6_^3−/4−^ solution containing 0.1 M KCl at a scan rate of 30 mV/s. **(B)** DPV curves obtained on different electrodes in mixture of AA (100 μM), DA (5 μM), and UA (5 μM).

The impact of electrochemical preactivation on electrode properties was examined using the standard redox probe Fe(CN)_6_^3−/4−^ through cyclic voltammetry (CV). As shown in Figure 2A, Fe(CN)_6_^3−/4−^ exhibits a distinct pair of oxidation/reduction peaks on GCE. After anodic polarization of GCE, there are almost no observable oxidation/reduction peaks on the obtained electrode, indicating that the high positive potential applied during anodic polarization imposes a strong oxidative effect on the electrode surface, inhibiting the electron transfer process at the electrode interface. Subsequently, after cathodic reduction of the electrode, oxidation–reduction peaks of Fe(CN)_6_^3−/4−^ molecules reappear on p-GCE. Compared to GCE, p-GCE demonstrates higher charging current and oxidation–reduction peak current, indicating an increased electrode active area. Moreover, the peak-to-peak separation (66 mV) on p-GCE is smaller than that on GCE (75 mV), suggesting faster charge transfer kinetics for the redox probe on p-GCE.

To study the effect of electrochemical preactivation on potential resolution of electrode, the electrochemical characteristics of common electrochemical small molecules found in biological and food samples, including ascorbic acid (AA), uric acid (UA), and dopamine (DA) on GCE and p-GCE were further investigated. Figure 2B illustrates the differential pulse voltammetry (DPV) curves obtained on GCE and p-GCE in the analysis of the mixture of the three substances. As shown, GCE is unable to differentiate between the three electroactive components, detecting only a single mixed peak with a lower peak current. In contrast, p-GCE is able to distinguish between the three components, exhibiting higher peak currents and superior potential resolution capabilities. This may be attributed to the catalytic effect of oxygen-containing functional groups on the surface of p-GCE, thereby enhancing the electrode’s potential resolution capability.

GCE cannot directly and stably anchor VMSF. Processes such as washing after VMSF growth resulted in film detachment. Studies have shown that pre-treating with an organosilane adhesive layer can achieve stable VMSF modification (55). However, this operation increases the complexity of electrode preparation and, on the other hand, a non-conductive adhesive layer can reduce the electrode’s electrochemical activity. After preactivation of GCE, the successful integration of VMSF on p-GCE could be achieved without the use of any adhesives. This may be attributed to the formation of-O-Si-O-covalent bonds between the oxygen-containing functional groups on the surface of p-GCE and VMSF. As shown, the electrochemical assisted self-assembly (EASA) method was employed on p-GCE to further integrate VMSF. Currently, the most common methods for VMSF growth are the Stöber solution growth method and EASA method. The former can achieve large-area VMSF preparation on indium tin oxide (ITO) electrode but requires a longer preparation time (over 12 h). On the other hand, EASA method allows for rapid VMSF preparation on various electrodes, typically within 10 s. Based on the size exclusion effect and enrichment of VB_2_ of VMSF nanochannels, direct and highly sensitive electrochemical detection of VB_2_ in complex samples could be realized.

### 3.2 Integrity, charge-selective permeability, and size exclusion effect of VMSF

**Figure 3:**
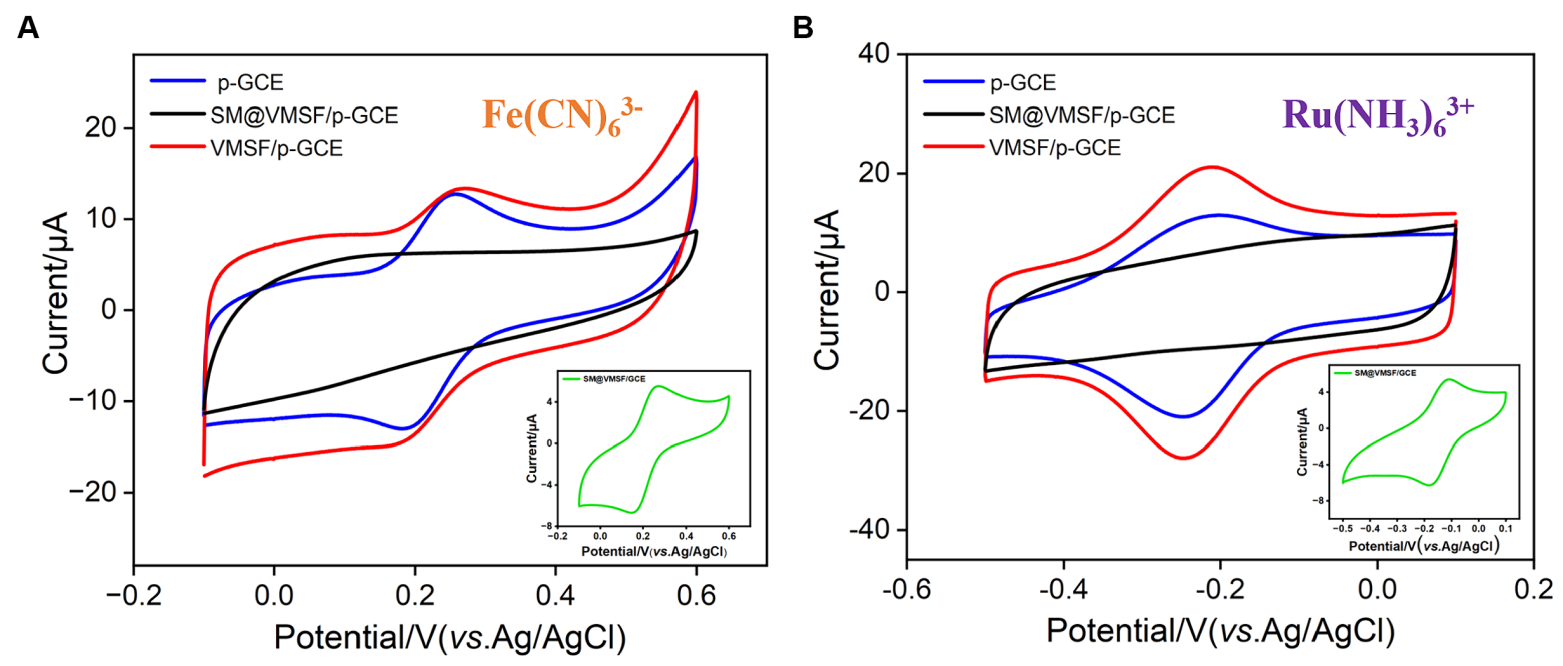
CV curves obtained on p-GCE, SM@VMSF/p-GCE, VMSF/p-GCE, and SM@VMSF/GCE (inset) in **(A)** K_3_Fe(CN)_6_ (0.5 mM) or **(B)** Ru(NH_3_)_6_Cl_3_ (0.5 mM) solution in KHP (0.05 M, pH = 7.4).

By examining the electrochemical performance of two standard redox probes with opposite charges on different electrodes, the integrity and charge-selective permeability of VMSF were investigated. Figures 3A,B show the CV curves obtained on p-GCE, SM@VMSF/p-GCE, and VMSF/p-GCE electrodes in solutions containing the anionic probe Fe(CN)_6_^3−^ or the cationic probe Ru(NH_3_)_6_^3+^, respectively. Both probes exhibit well-defined oxidation/reduction peaks on p-GCE. When SM@VMSF/p-GCE is used as the working electrode, almost no Faraday current is detected on the electrode. This is attributed to the non-conductive VMSF covering the electrode surface on p-GCE, and the nanochannels of VMSF are filled with SM, keeping nanochannels in a closed state. As a result, redox probe cannot reach the electrode surface through the nanochannels, confirming the integrity of the VMSF film. In marked contrast, when VMSF is directly grown on GCE, the resulting SM@VMSF/GCE exhibits significant oxidation–reduction peaks for both probes (insets in Figures 3A,B). This is attributed to the weak binding between GCE and VMSF, causing the film to detach during cleaning processes after growth, thereby failing to completely cover the electrode surface. This also proves the importance of electrode preactivation to achieve stable binding of VMSF without the need for adhesives. Upon removing SM to obtain the VMSF/p-GCE electrode, oxidation/reduction peaks for both probes can be observed again. Compared to p-GCE, the peak current of the negatively charged Fe(CN)_6_^3−^ probe on the VMSF/p-GCE electrode decreases, while the peak current of the positively charged Ru(NH_3_)_6_^3+^ probe significantly increases. This demonstrates the charge-selective permeability of the negatively charged surface of VMSF. The silica structure of VMSF is rich in silanol groups (p*K*a ~ 2), and their ionization in the buffer media results in a negatively charged surface. This leads to electrostatic repulsion toward negatively charged Fe(CN)_6_^3−^ and significant electrostatic attraction for positively charged Ru(NH_3_)_6_^3+^.

**Figure 4:**
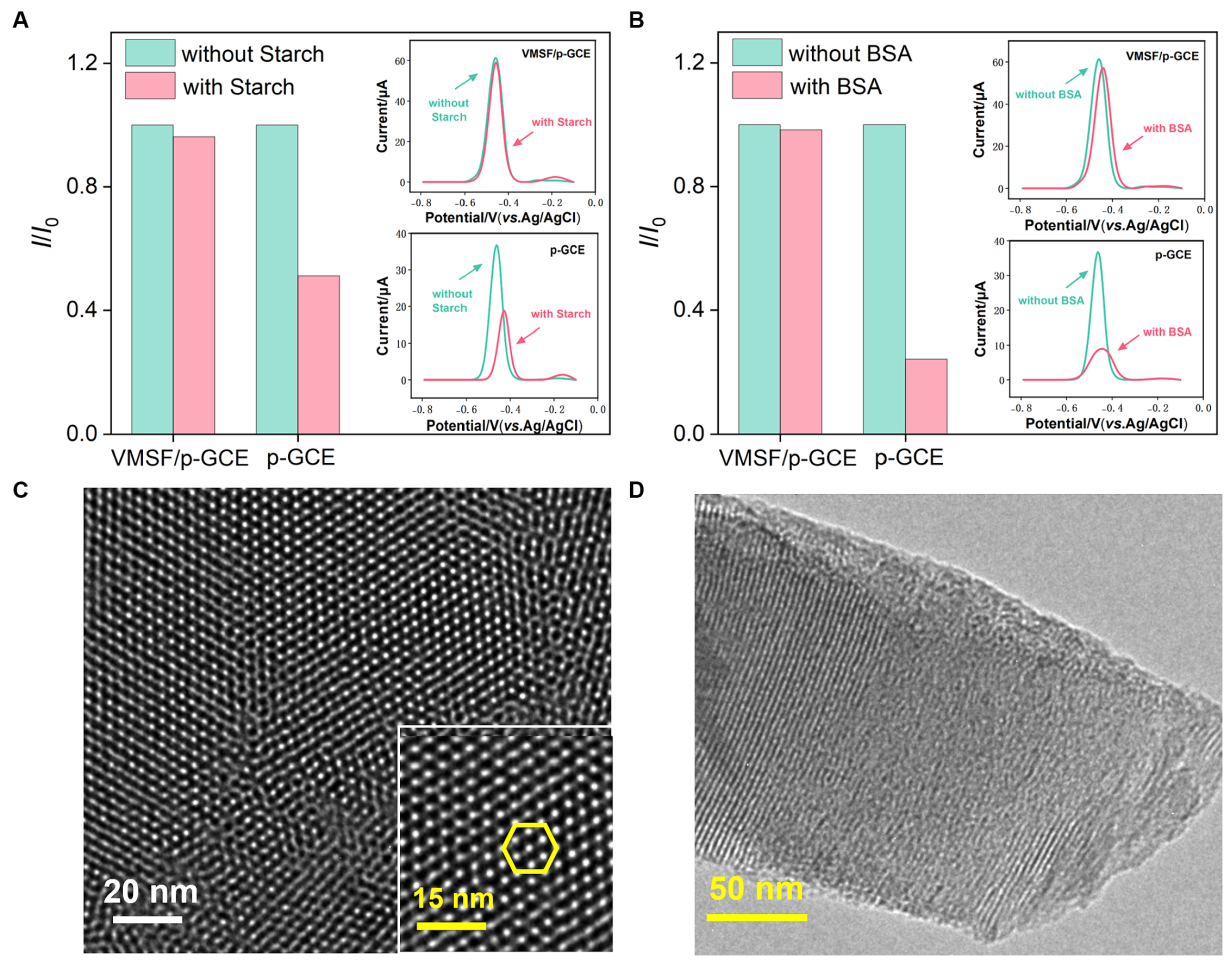
Stability of the VMSF/p-GCE. Normalized peak current ratio on VMSF/p-GCE or p-GCE toward VB_2_ (5 μM). *I* and *I*_0_ represent the currents obtained in the present and absence of 5 μg/mL of **(A)** Starch and **(B)** BSA in PBS (0.1 M, pH = 7). The insets are the corresponding DPV curves obtained for the p-GCE and VMSF/p-GCE in the absence and presence of starch or BSA. Top-view **(C)** and cross-sectional **(D)** TEM images of VMSF at different magnifications.

When detecting complex food or drug samples with an electrochemical sensor, large molecules in the matrix often non-specifically adsorb to the electrode surface, thereby contaminating the electrode surface and affecting the sensitivity and reproducibility of detection. Using VB_2_ as the electrochemical substance and bovine serum albumin (BSA) and starch as macromolecular models, the anti-fouling characteristic of p-GCE or VMSF/p-GCE electrodes were investigated. As shown in Figures 4A,B, when BSA or starch is present, the response of the p-GCE electrode to VB_2_ is significantly reduced, indicating that these substances tend to contaminate the electrode and reduce the detection signal. In contrast, when these macromolecules are present, the DPV peak current of VB_2_ obtained on the VMSF/p-GCE electrode remains almost unchanged, demonstrating the excellent anti-fouling capability. This anti-fouling ability is attributed to the size exclusion effect of the VMSF ultra-small nanochannels. Since these large molecules cannot enter the nanochannels, and the silica structure of VMSF is electrically insulating, BSA or starch can not contaminate the underlying electrode. This gives VMSF/p-GCE an advantage in the direct detection of complex samples.

### 3.3 Morphology characterization of VMSF

The morphology and structure of VMSF were characterized using transmission electron microscopy (TEM). As shown in Figure 4C, the TEM image of VMSF in a top-down view clearly reveals hexagonally arranged mesoporous nanochannels. The film appears uniform, with no observable defects over a large observed area. Figure 4D displayed the TEM image of the cross-sectional section of VMSF with ordered nanochannels with a film thickness (length of nanochannel) of approximately 120 nm.

### 3.4 Feasibility of electrochemical detection of VB_2_ and dual signal amplification

**Figure 5:**
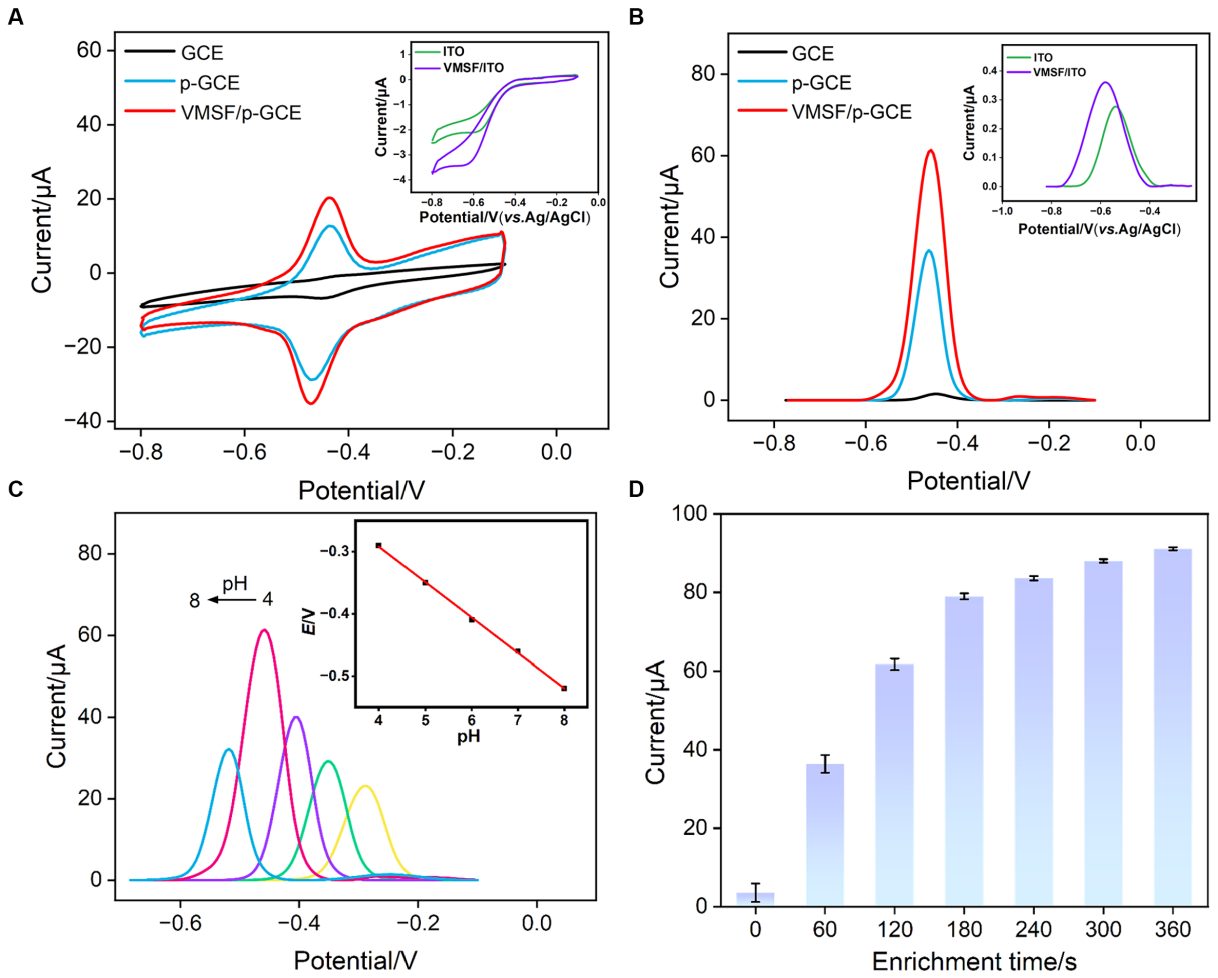
**(A)** CV curves and **(B)** DPV obtained on different electrodes in PBS (0.1 M, pH = 7.0) containing VB_2_ (5 μM). The scanning rate is 100 mV/s. The enrichment time is 180 s. Inset is the DPV obtained on ITO and VMSF/ITO under the same conditions. **(C)** CV curves obtained at 100 mV/s on VMSF/p-GCE in the presence of 5 μM VB_2_ in PBS (0.1 M) at various pH values. Inset is the linear dependence of *E*_pa_ on pH value. **(D)** The peak current obtained on VMSF/p-GCE at different accumulate time.

The electrochemical behavior of VB_2_ on different electrodes was investigated using CV and DPV. ITO electrodes and GCE electrodes were employed as controls. Figures 5A,B depict the CV and DPV curves obtained on various electrodes in VB_2_ solution. From the inset in Figure 5A, it can be observed that there are no apparent oxidation or reduction peaks for VB_2_ on the ITO electrode. Only very little peak current signal is observed in the corresponding DPV curve (inset in Figure 5B). These results indicates that the ITO electrode is not suitable as a supporting electrode for detecting VB_2_, an organic electroactive molecule. However, when GCE was used for detection, oxidation/reduction peaks of VB_2_ appear in the CV, and an oxidation peak near-0.47 V is detected in the DPV curve. This is attributed to the presence of aromatic structure in the VB_2_ structure, which can adsorb to the electrode surface through π-π stacking interactions, leading to an increase in peak current. For p-GCE obtained after electrode preactivation of GCE, both the oxidation–reduction peaks of VB_2_ in CV curve and the oxidation peak current in DPV curve are significantly enhanced. This is because electrochemical preactivation results in a larger electroactive surface area of the electrode, with abundant edge carbon, defects, and oxygen-containing functional groups that can further promote the adsorption and electron transfer reactions of the analyte on the electrode surface through hydrogen bonding or electrostatic interactions. Upon modification of VMSF on the surface of p-GCE, the peak current of VB_2_ measured on VMSF/p-GCE is further increased, demonstrating the significant enrichment effect of VMSF nanochannels on VB_2_. Despite the enrichment effect of nanochannels, the signal of VB_2_ on VMSF/ITO electrode is higher than that on ITO electrode but significantly lower than that obtained on VMSF/p-GCE. Thus, both the supporting electrode and the array of nanochannels on VMSF/p-GCE contribute to the enhanced electrochemical signal of VB_2_.

### 3.5 Optimization of electrochemical detection conditions for VB_2_

The growth thickness of VMSF on the surface of p-GCE might affect detection sensitivity of VMSF/ p-GCE. As the thickness of VMSF is related to the deposition time in growth (26), the electrochemical signals of electrodes obtained under different VMSF deposition time were investigated in VB_2_ solution. As shown in Supplementary Figure S2, the signal of VB_2_ increases when VMSF deposition time increases from 2 s to 10s. Subsequently, with a further increase in VMSF deposition time, the electrochemical signal decreases. It is speculated that an excessively thick VMSF layer may compromise the detection performance in two aspects. Firstly, when the VMSF thickness is too thick, the mass transfer of the analyte from the solution to the electrode surface takes a longer time. Secondly, with an excessively long growth time of VMSF, silica nanoparticles might form on the outer surface of VMSF, partially blocking the nanochannels.

To maximize the sensitivity of VB_2_ detection on VMSF/p-GCE, the detection conditions were optimized, including the pH of the supporting electrolyte and the VB_2_ enrichment time. As shown in Figure 5C, with the increase in pH, the DPV oxidation current of VB_2_ on the electrode gradually strengthened, reaching the maximum current response at pH = 7 (Figure 5C). Additionally, as the pH increased, the oxidation peak potential of VB_2_ shifted toward negative potentials, indicating the involvement of protons in the electrochemical oxidation–reduction process of VB_2_. From the inset in Figure 5C, it can be observed that the oxidation peak potential (*E*_pa_) of VB_2_ has a good linear relationship with pH (*E*_pa_ = −0.057 pH - 0.064, *R*^2^ = 0.9991). The slope is close to that of the Nernst equation (59 mV/pH), suggesting that the electrochemical oxidation–reduction process involving VB_2_ is an equivalent electron and proton transfer process. Thus, pH = 7 was chosen as the optimal detection condition. Under the optimal pH condition, time for VB_2_ enrichment was investigated (Figure 5D). With the increase in enrichment time, the DPV oxidation current of VB_2_ on the electrode gradually increased. After VB_2_ was enriched for 180 s, the change of current gradually decreased. Considering both the peak current and the detection speed, VB_2_ was enriched for 180 s for subsequent experiments.

**Figure 6:**
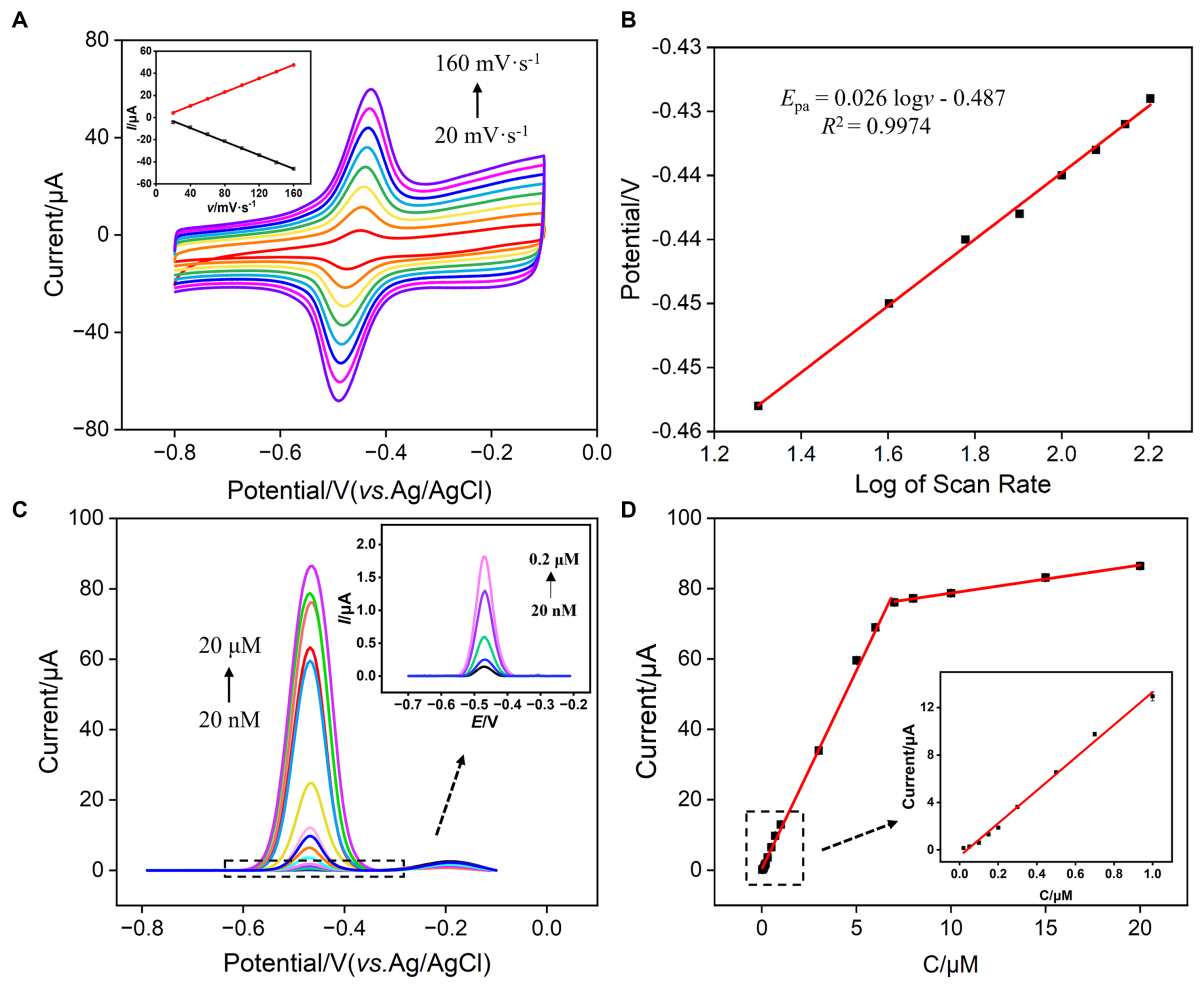
**(A)** CV curves obtained at different scan rates for VMSF/p-GCE in PBS (0.1 M, pH 7.0) containing 5 μM VB_2_. The inset is the dependence of *I*_pa_ on scan rate. The enrichment time is 180 s. **(B)** The dependence of the redox peak potentials of 5 μM VB_2_ on log of scan rate. **(C)** DPV curves obtained on VMSF/p-GCE in PBS (0.1 M, pH = 7.0) containing different concentrations of VB_2_. The inset is the magnified view of the DPV curves in the low concentration region. **(D)** The calibration curve for VB_2_. The inset in **(D)** shows the calibration lines in the low concentration range.

Faradaic charge is a crucial indicator of electrochemical substances undergoing oxidation–reduction reactions on electrodes. To study the charge transfer of VB_2_ on the sensing electrode, the relationship between the oxidation peak current and peak potential of VB_2_ with the scan rate (*ν*) was investigated. As shown in Figure 6A, with the increase in scan rate, the oxidation–reduction peak currents of VB_2_ gradually increased, and the peak potential shifted. The oxidation peak current (*I*_pa_) and reduction peak current (*I*_pc_) exhibited a good linear relationship with the scan rate (inset in Figure 6A, *I*_pa_ = 0.309*ν* − 1.77, *R*^2^ = 0.9999; *I*pc = −0.307*ν* + 2.86, *R*^2^ = 0.9988). This indicates that the electrochemical reaction process of VB_2_ molecules on the VMSF/p-GCE electrode is adsorption-controlled. The potential of oxidation peak (*E*_pa_) exhibits a well-defined linear relationship with the logarithm of the scanning rate (log*ν*) (Figure 6B), fitting to the following linear equation:


$$\begin{array}{l} E_{pa}={E_{0}}^{\prime }+m\left[0.78+\ln\left(\frac{D^{1/2}}{k_{s}}\right)-0.5\ln m\right]\\ \;+0.5\times 2.303\times \left(\frac{RT}{\alpha n_{\alpha }F}\right)\log v \end{array}$$

Here, *E_0_’* is the standard electrode potential, *k_s_* is the reaction standard rate constant, *D* is the diffusion coefficient of VB_2_, *α* is the electron transfer coefficient of VB_2_, and *n_α_* is the electron transfer number. The calculated *n_α_* is 2.28, approximately equal to 2, indicating that the reaction is a double electron transfer process. The redox reaction is illustrated in Figure 1.

### 3.6 Electrochemical detection of VB_2_

Under optimal conditions, the performance of VMSF/p-GCE in electrochemical detection of VB_2_ was investigated. Figure 6C illustrated the DPV curves obtained by VMSF/p-GCE in solutions with different concentrations of VB_2_. It can be observed that as the concentration of VB_2_ increases, the oxidation peak current gradually increases. As revealed in Figure 6D, the oxidation peak current (*I*) and VB_2_ concentration (*C*) exhibit a well-defined linear relationship in the range of VB_2_ concentrations from 20 nM to 7 μM (*I* = 11.3*C* + 0.401, *R*^2^ = 0.997), and from 7 μM to 20 μM (*I* = 0.797*C* + 70.8, *R*^2^ = 0.996). The calculated detection limit (LOD) is 11 nM at a signal-to-noise ratio of 3 (S/N = 3). Table 1 outlines the detection performance of various electrochemical sensors for VB_2_ (35–42). The LOD is lower than that obtained on nanoporous gold modified GCE (NPG/GCE) (35), reduced MoS_2_-graphene/32-merhomoadenine ssDNA oligonucleotides modified Au electrode (rMoS_2_-Gr/A_32_/AuE) (37), magnetite nanoparticles/reduced graphene oxide modified GCE (Fe_3_O_4_NPs/rGO/GCE) (38), Co^2+^-Y zeolite modified carbon paste electrode (Co^2+^-Y/CPE) (40), Fe_3_O_4_NPs modified electrochemical paper-based analytical devices (Fe_3_O_4_NPs-ePADs) (41), carbon cloth supported two-dimensional MoS_2_-MoO_3_ modified screen-printed carbon electrode (2D-MoS_2_-MoO_3_-CC/SPCE) (42), but higher than that obtained on α-Fe_2_O_3_ nanoparticles/multi-walled carbon nanotube/gold nanoparticles modified GCE α-Fe_2_O_3_NPs/MWCNT/AuNPs/GCE (36), vitamin B_2_-poly-O-aminophenol/molecularly imprinted polymers modified GCE (VB_2_-PoAP/MIPs/GCE) (39). The low LOD and high sensitivity, attributed to the dual enrichment effect of the supporting p-GCE and VMSF on VB_2_.

**Table 1:** Comparison of VB_2_ detection performance using different methods.

Electrode materials	Method	Detection range (μM)	LOD (nM)	Sensitivity (μA μM^−1^)	Step	References
NPG/GCE	DPV	5–250	100	0.178	1	Wang et al. (56)
α-Fe_2_O_3_NPs/MWCNT/AuNPs/GCE	SWV	0.3–60	6	–	4	Sumathi et al. (57)
rMoS_2_-Gr/A_32_/AuE	DPV	0.025–2.25	20	0.830	3	Wang et al. (58)
Fe_3_O_4_NPs/rGO/GCE	DPV	0.3–100	89	–	5	Madhuvilakku et al. (59)
VB_2_-PoAP/MIPs/GCE	DPV	0.01–0.12	2.38	–	3	Xu et al. (60)
Co^2+^-Y/CPE	CV	1.7–34	710	1.104	4	Nezamzadeh-Ejhieh et al. (61)
Fe_3_O_4_NPs-ePADs	SWV	2–20	250	4.87	4	Pereira et al. (62)
2D-MoS_2_-MoO_3_-CC/SPCE	LSV	2–40	1.5 × 10^3^	0.67	5	Zribi et al. (63)
VMSF/p-GCE	DPV	0.02–0.7 0.7–20	11	11.3 0.797	2	This work

NPG, nanoporous gold; α-Fe_2_O_3_NPs, α-Fe_2_O_3_ nanoparticles; MWCNT, multi-walled carbon nanotube; AuNPs, gold nanoparticles; rMoS_2_-Gr, reduced MoS_2_-graphene; A_32_, 32-merhomoadenine ssDNA oligonucleotides; AuE, Au electrode; Fe_3_O_4_NPs, magnetite nanoparticles; rGO, reduced graphene oxide; VB_2_-PoAP, vitamin B_2_-poly-O-aminophenol; MIPs, molecularly imprinted polymers; Co^2+^-Y, Co^2+^-Y zeolite; CPE, carbon paste electrode; ePADs, electrochemical paper-based analytical devices; 2D, two-dimensional; CC, carbon cloth; SPCE, screen-printed carbon electrode.

### 3.7 Selective, regenerative, and stable performance of the fabricated sensor

**Figure 7:**
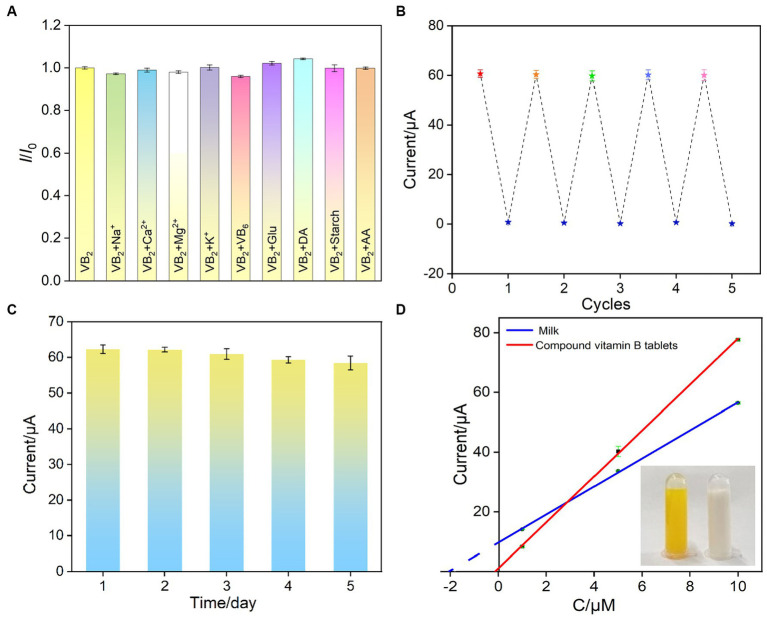
**(A)** The current ratio (*I*/*I*_0_) obtained on VMSF/p-GCE for detection of VB_2_ (5 μM) in the absence (*I*_0_) and presence (*I*) of 5 μM (Na^+^, Ca^2+^, Mg^2+^, K^+^, VB_6_, Glu, DA, Starch, AA) of other added interfering species. **(B)** The reuse performance of VMSF/p-GCE. The peak currents are obtained in electrolyte (bottom) or VB_2_ solution (top) using the regenerated electrodes. **(C)** Stability of the VMSF/p-GCE. The currents of the electrodes in the presence of VB_2_ (5 μM) over a 5-day storage period. After each measurement, the electrode was regenerated. **(D)** The standard curve for detection of VB_2_ in milk or leachate of compound vitamin B tablet using standard addition method. Inset is the image of leachate of compound vitamin B tablet (left) or milk (right) sample.

When the electrode was used, the complex matrix in real samples might affect the analysis. For instance, large molecules in samples, such as protein, DNA, polysaccharide, can nonspecifically adsorb and occupy active sites on the electrode surface, leading to electrode fouling. This fouling of this electrode will result in changes in electrochemical signals, reducing the accuracy of electrochemical detection. In addition, the co-existed redox molecules might also affect the detection. The selectivity and anti-fouling property of VMSF/p-GCE in detecting VB_2_ was investigated. Common metal ions (Na^+^, Ca^2+^, Mg^2+^, K^+^), large molecular, starch, and small molecules present in the human body including ascorbic acid-AA, uric acid-UA, vitamin B_6_, dopamine-DA, glucose-Glu were selected as the possible interfering substances to examine their impact on VB_2_ detection. As shown in Figure 7A, the addition of each above substance does not cause a significant change in the current signal of VB_2_, demonstrating the excellent selectivity of VMSF/p-GCE in VB_2_ detection. This is attributed to the distinct electrochemical potentials between the mentioned substances and VB_2_ on p-GCE. The electrostatic repulsion toward negatively charged redox molecules including AA and UA, also improve the selectivity. Additionally, large molecule such as starch cannot enter the nanochannel or contact with the underlying electrode. Thus, VSMF-modified electrode demonstrates anti-fouling property. To investigate the repeatability of VB_2_ detection among VMSF/p-GCE, five VMSF/p-GCE electrodes were prepared using different batches. The relative standard deviation (RSD) of the current signal for detecting VB_2_ (5 μM) was 0.8%, demonstrating good reproducibility in electrode preparation (Supplementary Figure S3).

The regenerative capability of VMSF/p-GCE was also examined. Stirring the electrode in hydrochloric acid-ethanol solution (0.1 M) for 20 min can effectively remove residual VB_2_ inside the channels, completing the electrode regeneration. After regeneration, the electrochemical signal measured in the supporting electrolyte was extremely low, indicating the effective removal of VB_2_ from the channels during the regeneration process (Figure 7B). The electrode was reused five times for VB_2_ detection after regeneration. Compared with the initial detection signal, the obtained peak currents are all higher than 98% with a relative standard deviation (RSD) of 0.5%, demonstrating excellent regenerative capability of the sensor.

The VMSF/p-GCE was stored at room temperature, and VB_2_ detection followed by regeneration was performed daily. It was observed in that even after regeneration and storage for 5 days, the peak current measured by VMSF/p-GCE remained at 93.8% of the first-day measurement, proving the electrode’s high stability (Figure 7C).

### 3.8 Real sample analysis

To assess the accuracy and precision of the sensor in real sample detection, the standard addition method was employed to detect the VB_2_ content in leachate of compound vitamin B tablet and milk samples. As shown in Table 2, the recovery rates ranged from 94.2 to 102%, with a RSD of less than 4.5% for three measurements, demonstrating the accuracy of the detection. Additionally, the VB_2_ content in the leachate of compound vitamin B tablet was determined using the extrapolation method and found to be 0.318 μM (as shown in Figure 7D), which closely aligned with the value determined by the standard (HPLC GB5009852016). Due to its convenient preparation, simple operation, and the absence of complex sample pretreatment, the developed electrochemical sensor has significant potential for direct electroanalysis of VB_2_ in complex samples.

**Table 2:** Determination of VB_2_ in leachate of compound vitamin B tablet or milk.

Sample	Added^a^ (μM)	Found (μM)	RSD (%, *n* = 3)	Recovery (%)
leachate of compound vitamin B tablet	0	0.318	2.5	–
	1.00	0.947	3.5	94.7
	5.00	5.09	4.5	101
	10.0	9.96	0.46	99.6
Milk	1.00	0.942	2.7	94.2
	5.00	5.10	3.4	102
	10.0	9.95	2.6	99.5

a The real samples were first spiked with the targets VB_2_ and then diluted with the supporting electrolyte solution by a factor of 100. The indicated concentration was obtained after dilution.

## 4 Conclusion

In summary, VMSF modified carbon-based electrode was fabricated through a simple and rapid method for highly sensitive detection of vitamin B_2_. Utilizing electrochemical preactivation of GCE, a p-GCE with excellent potential resolution and high electrochemical response was prepared. Using p-GCE as the supporting electrode, VMSF is directly grown via a rapid EASA method, eliminating the need for any adhesives and ensuring the stable integration of VMSF. Due to the dual enrichment of the supporting electrode and nanochannels, VMSF/p-GCE exhibited high sensitivity for electrochemical detection of VB_2_. Owing to the potential resolution of p-GCE and selectivity permeability of VMSF nanochannel, VMSF/p-GCE exhibits good selectivity and high anti-fouling performance. Direct electrochemical detection of VB_2_ in turbid samples without the need for complex sample pretreatment is realized. The sensor constructed in this study has advantages including simple fabrication, high sensitivity, good selectivity and excellent regeneration, holds promising applications in direct electroanalysis of VB_2_ in real samples. If miniaturized electrodes or electrochemical detection devices controlled by smartphones are used, portable VB_2_ detection is expected to be carried out.

## Data availability statement

The original contributions presented in the study are included in the article/Supplementary material, further inquiries can be directed to the corresponding author.

## Author contributions

YW: Data curation, Investigation, Writing – original draft. ZS: Data curation, Investigation, Writing – original draft. JL: Writing – review & editing. TL: Investigation, Writing – review & editing. FX: Conceptualization, Supervision, Writing – review & editing. QZ: Conceptualization, Writing – review & editing.
