# Supplementary material for: Simple fabrication of electrochemical sensor based on integration of dual signal amplification by the supporting electrode and modified nanochannel array for direct and sensitive detection of vitamin B2
Source: Front Nutr. 2024 Mar 15;11:1352938. doi: 10.3389/fnut.2024.1352938 (PMC10978690; doi:10.3389/fnut.2024.1352938)
Supplement: Supplementary file 1 [file Image_1.pdf]

Supplementary Information

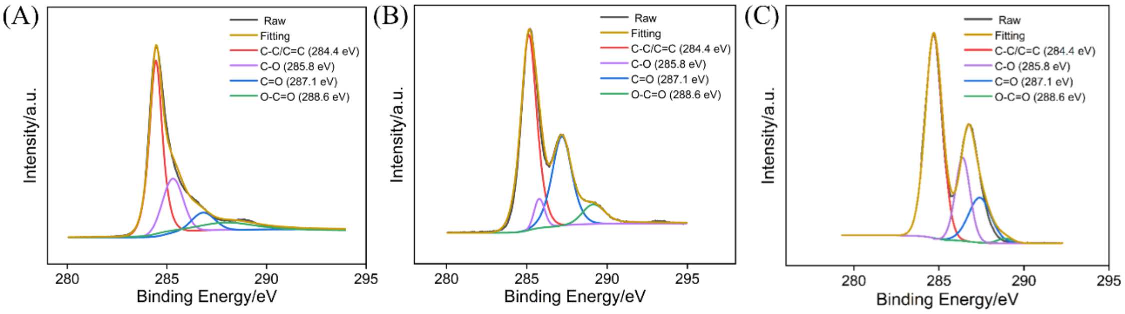

Figure S1 High-resolution XPS C1s spectra of GCE (A), the electrode after anodic polarization (B), and p-GCE (C).

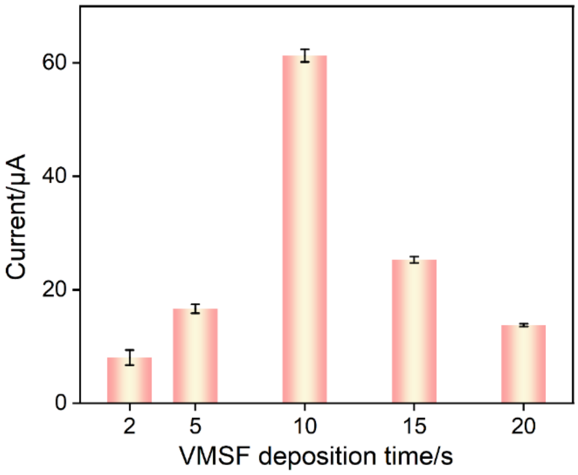

Figure S2. The peak current of VB_2 (5 μM) obtained on VMSF/p-GCE fabricated at different VMSF deposition time.

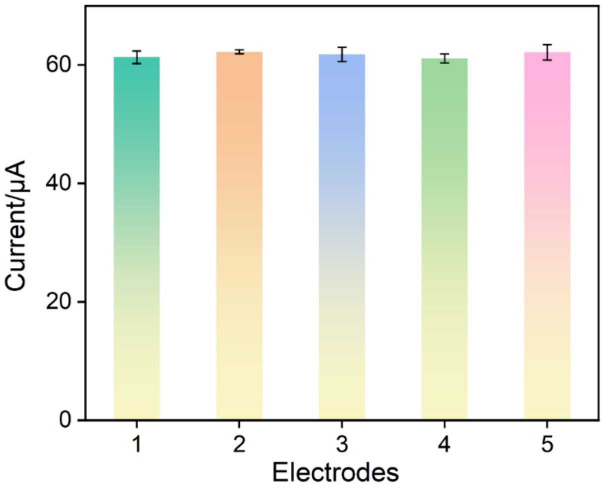

Figure S3. The peak current of VB_2 (5 μM) obtained on VMSF/p-GCE fabricated using different batches.
